# Immunomodulatory activity of semen Ziziphi Spinosae protein: a potential plant protein functional food raw material

**DOI:** 10.1038/s41538-023-00204-3

**Published:** 2023-06-19

**Authors:** Hongyin Zhang, Fengqin Xiao, Jia Li, Rongxin Han, Guangzhe Li, Zhiqiang Wan, Shuai Shao, Daqing Zhao, Mingming Yan

**Affiliations:** 1 Changchun University of Chinese Medicine, Changchun, Jilin China; 2 Jinlin Provincial Science and Technology Innovation Center of Health Food of Chinese Medicine, Changchun University of Chinese Medicine, Changchun, Jilin China

**Keywords:** Immunology, Nutrition

## Abstract

Semen Ziziphi Spinosae protein (SZSP) is a new plant protein resource with good food functional properties and health care function. However, the biological activity of SZSP has not been further studied, which greatly limits the development and utilization of SZSP in the food industry. The aim of this study was to investigate the protective effect of SZSP on immunosuppressed mice and its inhibitory effect on immune-stimulated RAW264.7 cells. The results demonstrated that SZSP remarkably improved the immunomodulatory secretion in serum (interleukin-2, tumor necrosis factor-α [TNF-α], interferon-γ, immunoglobulin-A, immunoglobulin-G, immunoglobulin-M) and primary macrophages (nitric oxide, interleukin-1β, TNF-α) and promoted the NK-cell killing activity of primary splenocytes in CTX-induced immunosuppression mice. Immunohistochemical analysis results indicated that the secretion of CD_4_^+^ and CD_8_^+^ in the spleen and thymus can be regulated by SZSP, leading to inhibition of the damage induced by cyclophosphamide in mice. Meanwhile, in order to clarify the immunomodulatory mechanism of SZSP, we showed that SZSP significantly inhibited the secretion of NO, interleukin-6, and TNF-α and reduced the phosphorylation expression of p-ERK, p-JNK, and p-IκBα in lipopolysaccharide-stimulated RAW264.7 cells. Therefore, the immunomodulatory effect of SZSP may be related to the activation of MAPKs and NF-κB signaling pathways. Based on the above studies, the preliminary purification of SZSP was continued, and S1F2G1 with immunomodulatory activity was obtained. Taken together, SZSP has an immunoregulatory effect in vivo and in vitro and may be a favorable candidate of functional food raw material for regulating immune responses.

## Introduction

Food resources with immunomodulatory potential are favored in research on healthy food because their daily consumption can improve immune-regulation ability, reduce the decline in body functions, improve quality of life, and reduce the occurrence of diseases^1,2^. The main function of the human immune system is to prevent diseases by identifying and killing harmful pathogens and tumor cells^3^. Congenital or acquired damage to the body leads to structural or functional abnormalities of the immune system, which eventually leads to immune diseases^4^. The immune system is responsible for maintaining the immune homeostasis of the body under normal physiological conditions. The imbalance of immune homeostasis usually recovers and balances the immune environment of the body through the two-way regulation of immune stimulation and immunosuppression^5^. Specifically, immune stimulation is the positive regulation of the immune system to improve the immune response level of the body, secrete more immune factors, and achieve the effect of removing foreign invasion. It is mainly used to alleviate the low immune function caused by common autoimmune diseases, long-term use of immunosuppressants during tumor treatment, and congenital immune defects^6^. Immunosuppressive regulation refers to the negative regulation of immune response to inhibit the abnormal state of the body, usually to reduce the vitality or effect of the immune system, reduce the secretion of immune factors, weaken and even terminate the immune response, and prevent the occurrence of excessive immunity^7^. It is mainly used to alleviate autoimmune diseases caused by the abnormal sensitivity and overreaction of the immune system of the body and the attack of its own substances as invading foreign bodies, such as in rheumatoid arthritis and systemic lupus erythematosus^8,9^. Many natural ingredients, such as proteins, polypeptides, and polysaccharides, have attracted attention due to their immunomodulatory properties^10,11^. Because they are food-derived natural plant ingredients, especially with dual properties of food processing and biological regulation, they are more conducive to intake by the body^12^. Therefore, it is particularly important to find safe and stable immunomodulators without side effects to improve disease prevention and maintain health.

Recently, increasing attention has been paid to the regulation of immune function by natural protein components through triggering immune effector cells (such as natural killer cells and macrophages). The development of functional plant protein food supplements with immunomodulatory effects has been greatly encouraged. With the deepening of research, proteins that can regulate the body’s immune function have been obtained from various edible animal and plant products, such as milk, egg white, soybeans, mung beans, and other common foods^13–15^. In addition, proteins with obvious biological activity can be extracted from most food-derived medicinal plants, such as maca root, ginseng, peony seed, *Schisandra chinensis*, and cucumber seed^16–18^. Therefore, natural proteins from medicinal plants are an important resource for the development of natural immune regulators.

Semen Ziziphi Spinosae (SZS, the seeds of *Ziziphus jujuba* Mill. var. *spinosa* (Bunge) Hu ex H. F. Chou) is used as a traditional Chinese medicine referred to as Suan Zao Ren, and it is one of the most popular seeds of Rhamniaceae^19^. It can be eaten daily and is often used to cook porridge and make tea. It is often exported to Europe, the United States, and Southeast Asian countries as raw food material. SZS is a commonly used health food raw material and is one of the “medicine and food homologous” species, because of its role in regulating sleep, depression, and anxiety. SZS contains more than 25% of protein components. In our previous study, we showed that semen Ziziphi Spinosae protein (SZSP) extracted from SZS was rich in amino acid composition, and its content of essential amino acids meets the requirements of the World Health Organization/Food and Agriculture Organization of the United Nations for adult intake. In addition, it has excellent food processing properties such as solubility, thermal stability, and gel properties^20^. Pharmacological research results have shown that SZSP has a good scavenging capacity for oxygen free radicals and anti-fatigue effects^21^. It is a potential raw material for processing new plant proteins in the food industry.

The immunoregulatory activity of proteins is usually evaluated by combining different immune indicators in vivo and in vitro. For in vivo studies, cyclophosphamide (CTX) is often used as a model drug to establish immunosuppression because of its significant immunosuppressive effect^22^. CTX is a broad-spectrum alkylating-agent antitumor drug and is one of the most powerful and widely used immunosuppressants at present^23^. Similar to other chemotherapy drugs, long-term use of CTX causes serious immunosuppressive side effects and increases the probability of infection with bacteria and fungi^24^. CTX can reduce the secretion of immunoglobulins and immune factors in the blood and tissues of model animals, destroy the physiological structure of immune organs, and significantly reduce the weight of model animals^25^. Therefore, active substances that can inhibit the side effects of CTX are generally considered to have good immunomodulatory effects. Macrophages, immune defense cells, play an indispensable role in the process of immune regulation^26^. Lipopolysaccharide (LPS)-stimulated RAW264.7 macrophages are a commonly used in vitro model for studying immune stimulation. RAW264.7 cells stimulated by LPS can produce a variety of immune-regulatory mediators (iNOS) and a series of cytokines (such as IL-1β, IL-6, and TNF-α)^27^. LPS can also trigger a series of immune-regulatory signaling pathways, including nuclear factor κB (NF-κB) and mitogen-activated protein kinases (MAPKs)^28^. Therefore, the exploration of immune regulation mechanism can be realized by regulating the abovementioned signaling pathways.

In the present study, the immunoregulatory activity of SZSP on CTX-induced mice and LPS-stimulated RAW264.7 cells was examined through histomorphological and biochemical methods, and the effects of different doses of SZSP on macrophages, lymphocytes, NK cells, and serum hemolysin in mice were analyzed. The immunomodulatory mechanism of SZSP on RAW264.7 cells was analyzed by western blot analysis through the NF-κB and MAPKs signaling pathways. These results may contribute to the further development of SZSP resources and provide data and experimental support for immune-enhancing foods and health foods.

## Results

### Effects of SZSP obtained by different extraction methods on the activity of RAW264.7 cells

The common methods of protein extraction from plant food include ammonium sulfate precipitation (AS), aqueous extraction (AE), alkali extraction, and acid precipitation (AA). Depending on the structural characteristics of proteins, different protein components are obtained according to their solubility and pH value in different solvents. The research on protein activity mainly involves the protein active components obtained by the AS method, which may be because they are extracted at low temperatures to effectively prevent protein denaturation. We compared the effects of SZSP obtained by the three extraction methods, namely AE-SZSP, AS-SZSP, and AA-SZSP, on the immune activity of RAW264.7 cells (Table 1). We found that AS-SZSP and AA-SZSP had better immunomodulatory activity; however, the extraction rate of AA-SZSP was significantly higher than the other two proteins. In 100 g skimmed powder, AS-SZSP accounted for 15.23 ± 2.45 g/100 g, AE-SZSP for 20.31 ± 3.55 g/100 g, and AA-SZSP for 37.65 ± 3.41 g/100 g. SDS-PAGE showed that AA-SZSP was clear, and there were obvious bands at 15, 25, 35, and 40 kDa. Obvious protein subunit bands could not be observed in AS-SZSP and AE-SZSP. The analysis of immunoreactive factor secretion showed that AS-SZSP and AA-SZSP had effects on NO and TNF-α in LPS-stimulated RAW264.7 cells; AE-SZSP had a certain inhibitory effect, but its intensity was weak. We combined various factors to score and compare the proteins obtained by the three methods. Although the biological activity of AA-SZSP was slightly weaker than that of AS-SZSP, the yield of AS-SZSP was too low, which would affect the development of industrialization research from the perspective of food production. Therefore, this study suggested to carry out research on the biological activity of functional food based on AA-SZSP.

**Table 1:** Effect of semen Ziziphi Spinosae (SZS) protein on the extraction rate of defatted SZS flour, cell proliferation rate, and release of NO and TNF-α in LPS-stimulated RAW264.7 cells.

Groups	Extraction rate (g/100 g)	Cell proliferation rate (%)	NO (μmol/L)	TNF-α (pg/mL)
Control	–	100.00 ± 2.34	15.21 ± 0.21	720.19 ± 53.61
LPS	–	102.34 ± 4.56	59.75 ± 2.33^##^	3828.67 ± 99.84^##^
AS-SZSP	15.23 ± 2.45	118.37 ± 3.21	21.07 ± 2.76**	1185.62 ± 62.79**
AA-SZSP	37.65 ± 3.41	105.96 ± 2.04	22.81 ± 1.09**	1290.57 ± 37.25**
AE-SZSP	20.31 ± 3.55	111.36 ± 1.88	36.27 ± 1.24**	1968.78 ± 55.21**

Compared with the control group, ^##^*p* < 0.01; Compared with the LPS group, **p < 0.01.

### Effect of SZSP on body weight and organ index of CTX-induced mice

**Fig. 1:**
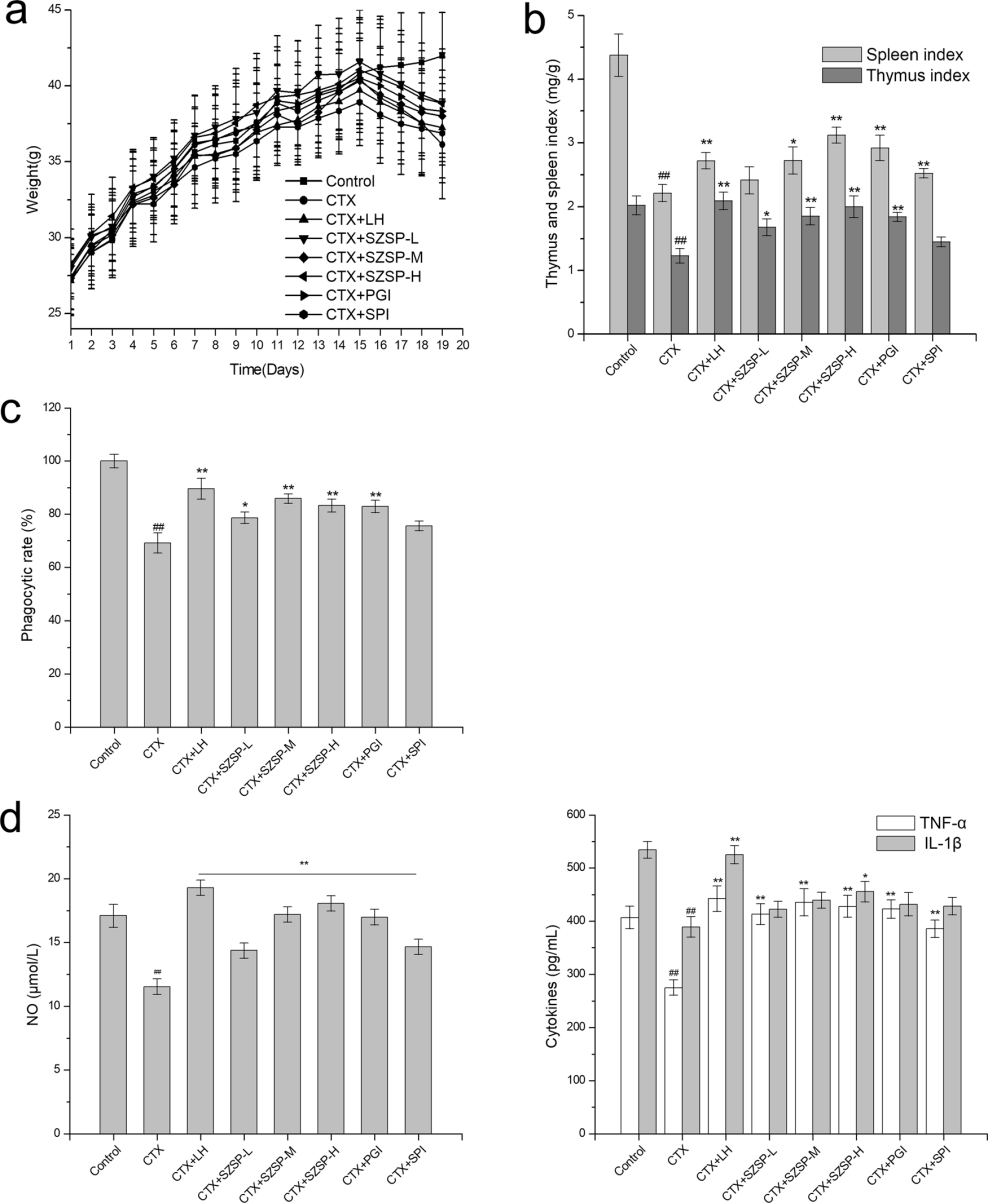
Effects of semen Ziziphi Spinosae protein (SZSP) on body weight, organ index, and primary macrophages in the CTX-induced immunosuppressive mice model. **a** Changes in body weight of mice during oral administration. **b** Organ index of the immunocompromised mice. **c** Phagocytic activity of primary macrophages. **d** Secretion of NO, TNF-α, and IL-6 in primary macrophages. Data are expressed as mean ± SD (*n* = 10). The doses of SZSP-L, SZSP-M, and SZSP-H were 100, 200, and 400 mg/kg bw/d, respectively. Compared with the control group, ^#^*p* < 0.05 and ^##^*p* < 0.01. Compared with the model group, *p < 0.05 and **p < 0.01.

To confirm the effect of SZSP on body weight changes of mice, the daily body weight of the mice was strictly recorded and analyzed (Fig. 1a), and the organ index of CTX-induced mice was detected (Fig. 1b). As shown in Fig. 1, the body weight of all mice showed an upward trend from day 1 to day 15, but the body weight in the CTX group decreased on day 16. The body weight and organ index in the CTX group were significantly lower than those in the control group, and the body weight in the control group increased normally, indicating that the growth of the mice was inhibited after intraperitoneal injection of CTX. The SZSP, LH, and PGI groups had a certain inhibitory effect on weight loss caused by CTX, but there was no significant difference between these groups and the model group (*p* > 0.05). As shown in Fig. 1b, the thymus and the spleen indexes of the CTX group mice were significantly lower than those of the control group (*p* < 0.05), meaning that CTX could significantly inhibit immune organs and weaken the immune function of mice. Compared with the model group, the indexes of the thymus and spleen in the treatment group were significantly higher. These results suggest that administration of SZSP before CTX can effectively inhibit the detrimental effects of CTX on mice.

### Effect of SZSP on peritoneal macrophages in CTX-induced mice

The phagocytic function of peritoneal macrophages is considered an important indicator of improving immune function. As shown in Fig. 1c, after intraperitoneal injection of CTX, the uptake of neutral red by primary peritoneal macrophages of mice was significantly inhibited. Compared with the CTX group, the phagocytic function of macrophages was significantly improved in the SZSP-M, SZSP-H, LH, and PGI groups (*p* < 0.01). In addition, the phagocytic function in the SZSP-M and PGI groups was close to that in the LH group, and the effect of SZSP was slightly better.

After external stimulation, macrophages secrete a variety of cytokines, such as TNF-α and IL-1β, to coordinate the immune response. To evaluate the effect of SZSP on CTX-induced murine peritoneal macrophages secreting immune regulatory factors, ELISA was performed (Fig. 1d). The results showed that CTX induced a decrease in the levels of NO, TNF-α, and IL-1β in peritoneal macrophages of the mice (*p* < 0.01). After administration, different SZSP groups and positive control groups showed certain promotive effects on the secretion of NO, TNF-α, and IL-1β. Among them, SZSP showed a significant effect on the secretion of NO and TNF-α (compared with the CTX group, *p* < 0.01), which was dose-dependent, but had a weak effect on the secretion of IL-1β (*p* < 0.05). In addition, among the three positive control groups, the LH group had a better effect than other administration groups, and the effect in the SPI group was slightly worse. However, we found that protein components had no significant effect on IL-1β in primary mouse macrophages. Together with the results of the phagocytosis, it may be concluded that SZSP can reduce CTX-related inhibition of primary peritoneal macrophages in mice.

### Effect of SZSP on humoral immunity in CTX-induced mice

**Fig. 2:**
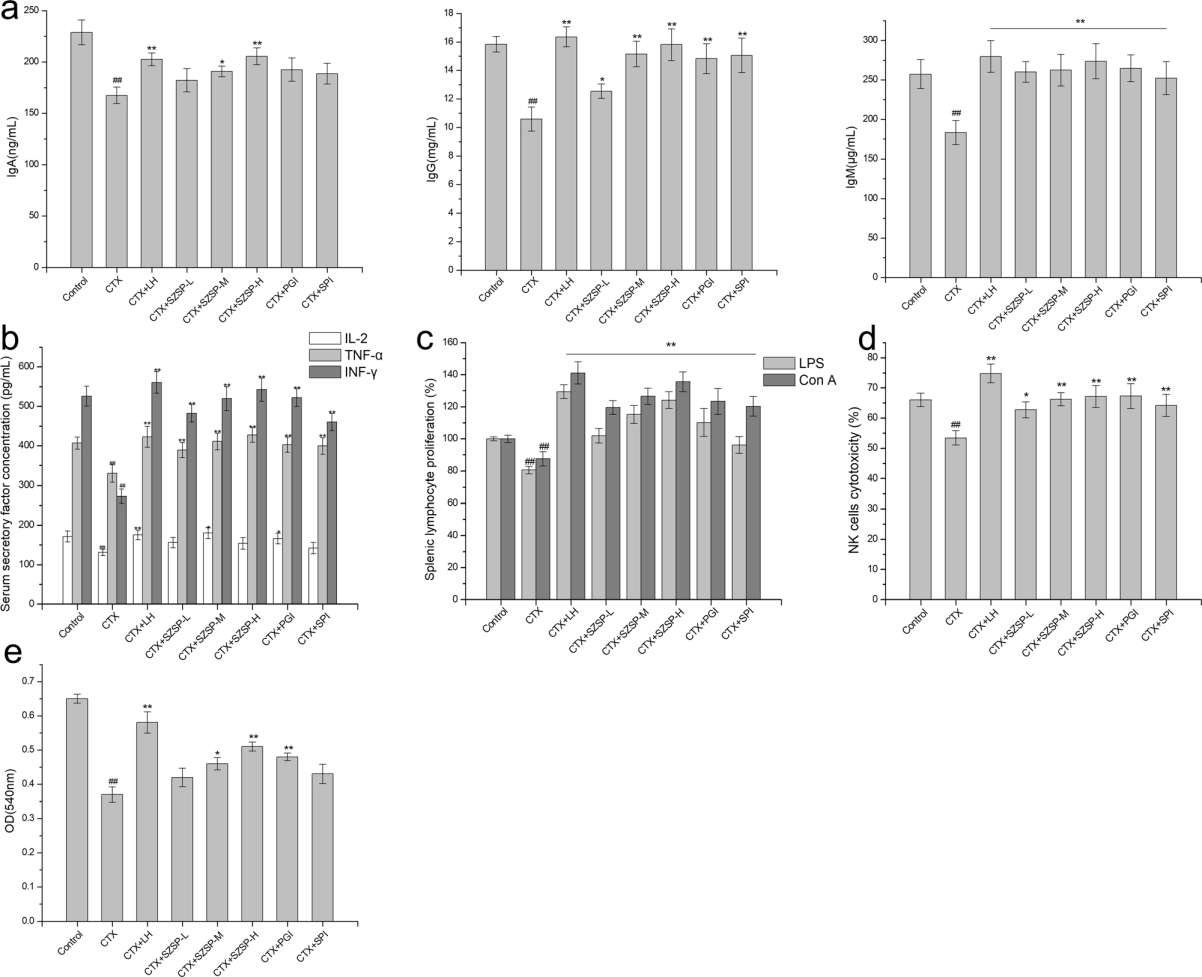
Effects of semen Ziziphi Spinosae protein (SZSP) on serum factors, primary splenocytes, NK-cell activity, and humoral immunity in the CTX-induced immunosuppressive mice model. **a** Immunoglobulin factor release in immunosuppressive mice serum. **b** Cytokine release in immunosuppressive mice serum. **c** Splenic lymphocyte proliferation by LPS and Con A induction in immunosuppressive mice. **d** NK-cell cytotoxicity in immunosuppressive mice. **e** Serum hemolysis level in immunosuppressive mice. Data are expressed as mean ± SD (*n* = 10). The doses of SZSP-L, SZSP-M, and SZSP-H were 100, 200, and 400 mg/kg bw/d, respectively. Compared with the control group, ^#^*p* < 0.05 and ^##^*p* < 0.01. Compared with the model group, *p < 0.05 and **p < 0.01.

To determine the effect of SZSP on humoral immunity, the levels of IgA, IgG, and IgM (Fig. 2a) and the concentrations of IL-2, TNF-α, and IFN-γ (Fig. 2b) in the serum of CTX-induced mice were detected. The effect of SZSP on immunoglobulin in mouse serum is shown in Fig. 2a. The serum levels of IgA, IgM, and IgG in the CTX group were significantly lower than those in the control group (*p* < 0.01). Compared with the CTX group, the SZSP and other protein administration groups showed significantly increased production of IgM and IgG (*p* < 0.01). As for the secretion of IgA, the effect of protein components was only significant in the SZSP-H group (*p* < 0.01), indicating that SZSP had a certain protective effect against CTX-induced immunosuppression.

As shown in Fig. 2b, the levels of IL-2, TNF-α, and IFN-γ were significantly reduced after CTX induction. The concentrations of IL-2, TNF-α, and IFN-γ declined from 171.46 pg/mL to 131.20 pg/mL, from 406.73 pg/mL to 330.17 pg/mL, and from 525.93 pg/mL to 272.99 pg/mL, respectively. In the SZSP group, IL-2, TNF-α, and IFN-γ levels were best restored to 154.10 pg/mL, 427.68 pg/mL, and 542.29 pg/mL, respectively, in a dose-dependent manner. In addition, the PGI and SPI groups had significant recovery effects on TNF-α and INF-γ (*p* < 0.01). The effect of the PGI group on IL-2 was similar to that of the SZSP-M group, but that of the SPI group was weaker than that of SZSP-L. The common results showed that SZSP was able to reverse the decrease in immunoglobulins and immune factors in the serum of the model mice, indicating that SZSP has the ability to regulate the secretion of various immune factors in humoral immunity, thereby enhancing immunity.

### Effect of SZSP on splenic lymphocytes in CTX-induced mice

The proliferation of T and B lymphocytes is an important index of the immune response system. Con A and LPS could induce the proliferation of T lymphocytes and B lymphocytes, respectively^29^. After co-culture with splenic lymphocytes for 24 h, the proliferative capacity of differentiated T lymphocytes and B lymphocytes was detected as an important index for evaluating the immune-regulatory effect. The results are shown in Fig. 2c. Compared with the control group, the proliferation activity of lymphocytes decreased significantly after intraperitoneal injection of CTX (*p* < 0.01). However, SZSP reversed this downward trend, and the differentiation of T lymphocytes and B lymphocytes increased significantly in the treatment group. SZSP-H effectively increased the proliferation of T lymphocytes induced by Con A, and B lymphocytes induced by LPS (*p* < 0.01), which was not significantly different from the LH group (*p* > 0.05). In addition, the proliferation rate of the SZSP-H group was slightly higher than that of the PGI group, but there was no significant difference (*p* > 0.05). Hence, SZSP has the potential to enhance the damaged immune system.

NK cells are the key cells in the innate immune system. When recognized and activated by target cells, NK cells can directly remove the cells infected with pathogens by activating and inhibiting the signal integration of receptors^30^. Figure 2d shows the effects of SZSP on NK-cell lethality in CTX-induced immunosuppressive mice. Compared with the control group, NK-cell activity in the CTX group decreased significantly (*p* < 0.01), and in the SZSP and LH groups, the activity of NK cells was enhanced, indicating that SZSP and LH can improve the cellular immune function of mice. The NK-cell activity recovered better in the SZSP-M and SZSP-H groups, but there was no significant difference between them (*p* > 0.05). The results of the PGI and SPI groups showed that the NK-cell activity of spleen lymphocytes was also increased (*p* < 0.05). These results show that long-term oral administration of SZSP can prevent CTX-induced immunosuppression, further indicating that SZSP has a potential immunomodulatory activity.

### Effect of SZSP on serum hemolysin level

To verify the effect of SZSP on the humoral immune response in mice, the changes in serum hemolysin in different administration groups were measured following SRBC injection. As shown in Fig. 2e, compared with the control group, CTX-induced serum hemolysin production was significantly inhibited in each group (*p* < 0.01). In contrast, after long-term oral administration of SZSP, PGI, and SPI, the CTX-induced inhibitory effect on serum hemolysin level gradually decreased. There was no significant difference between the SZSP-L and CTX groups (*p* > 0.05), but the trend improved with the increase of dosage. In addition, PGI had a good effect on the secretion of serum hemolysin in mice. In conclusion, SZSP treatment increased the level of serum hemolysin, suggesting that SZSP could enhance humoral immune activity.

### Effect of SZSP on H&E and immunohistochemistry findings of thymus in CTX-induced mice

**Fig. 3:**
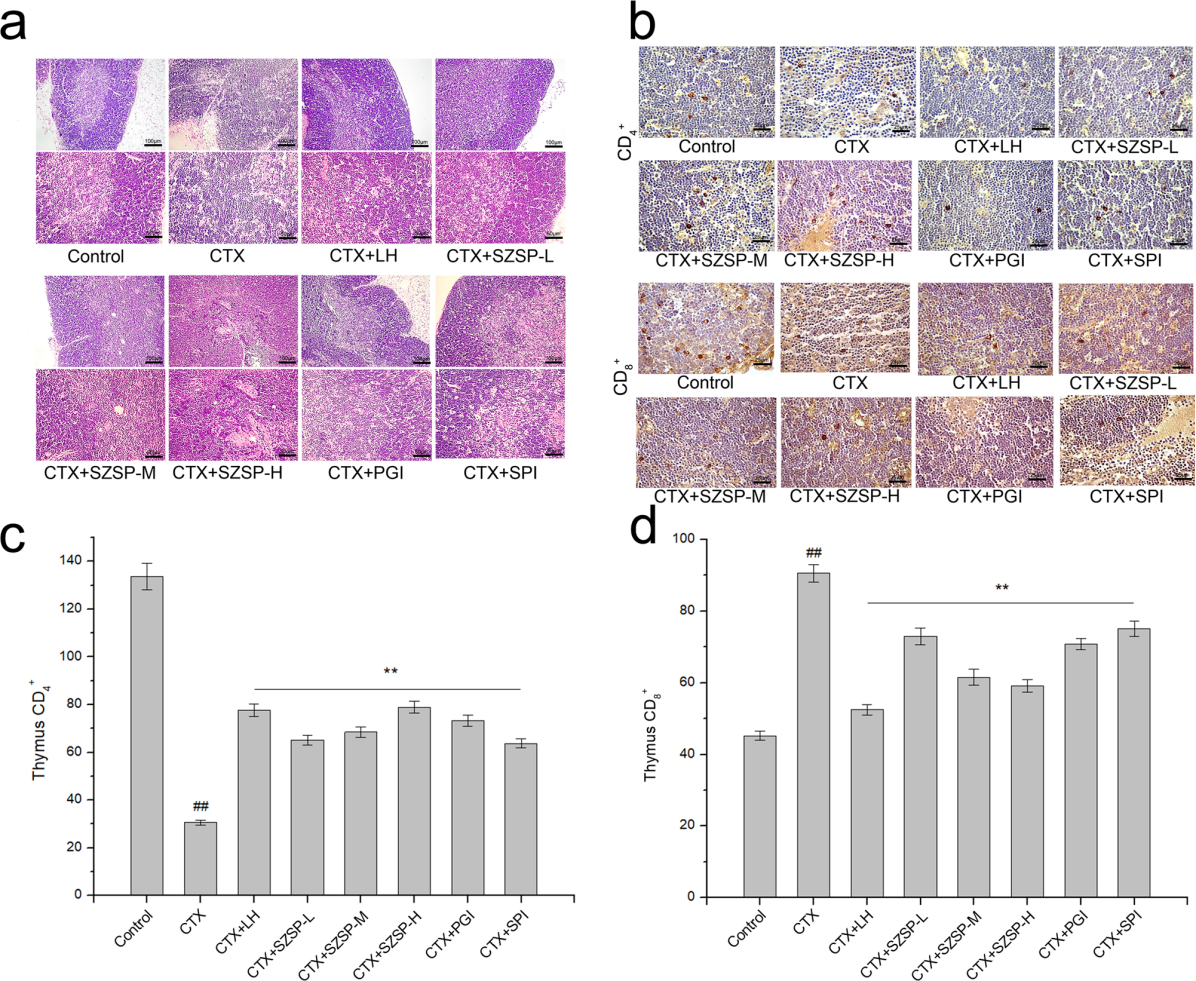
Thymus histopathology of CTX-induced immunosuppression mice. **a** H&E staining of the thymus in immunosuppressive mice. Scale bars, 100 and 50 μm. **b** Immunohistochemical analysis of the thymus in immunosuppressive mice. Scale bars, 25 μm. **c** Changes in CD_4_^+^ expression in the thymus of immunosuppressive mice. **d** Changes in CD_8_^+^ expression in the thymus of immunosuppressive mice. Data are expressed as mean ± SD (*n* = 10). The doses of SZSP-L, SZSP-M, and SZSP-H were 100, 200, and 400 mg/kg bw/d, respectively. Compared with the control group, ^#^*p* < 0.05 and ^##^*p* < 0.01. Compared with the model group, *p < 0.05 and **p < 0.01.

The histopathological analysis of the H&E-stained thymus is shown in Fig. 3a, b. Compared with the control group, the model group showed significant atrophy, cortical thinning, and medullary expansion of the thymus, and lymphocytes were irregularly round. Compared with the model group, the state of thymic lobules was better and the boundary between the cortex and medulla was clear in the SZSP-L (-M, -H), LH, PGI, and SPI groups. The number of lymphocytes increased, and the lymphocytes were round. Hence, the results showed that SZSP and plant proteins effectively prevented CTX damage to the mouse thymus.

The results of thymus immunohistochemistry showed that the expression level of CD_4_^+^ in thymocytes of the model group was significantly lower than that in the control group. After long-term oral administration of LH, SZSP, PGI, and SPI, the expression level of CD_4_^+^ in the thymus increased significantly. In addition, compared with the control group, the expression level of CD_8_^+^ in thymocytes of the model group significantly increased after being stimulated by CTX. After oral administration, the expression level of CD_8_^+^ in thymocytes of mice in each group decreased (Fig. 3b), and SZSP-H had the best effect. These results indicate that long-term oral administration of SZSP can effectively prevent the immunosuppressive effect of CTX on mice.

### Effect of SZSP on H&E and immunohistochemistry findings of spleen in CTX-induced mice

**Fig. 4:**
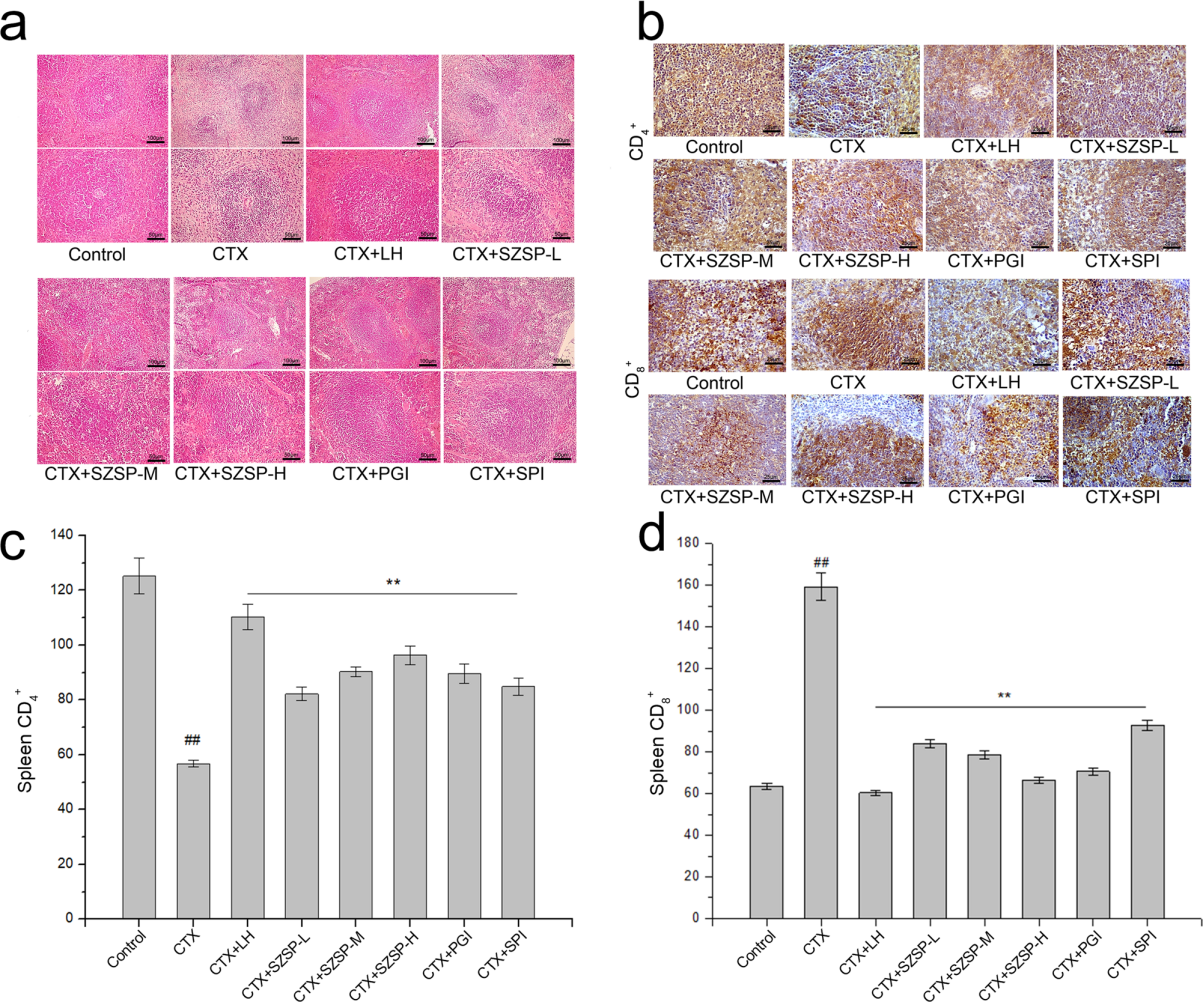
Spleen histopathology of CTX-induced immunosuppression mice. **a** H&E staining of the spleen in immunosuppressive mice. Scale bars, 100 and 50 μm. **b** Immunohistochemical analysis of the spleen in immunosuppressive mice. Scale bars, 25 μm. **c** Changes in CD_4_^+^ expression in the spleen of immunosuppressive mice. **d** Changes in CD_8_^+^ expression in the spleen of immunosuppressive mice. Data are expressed as mean ± SD (*n* = 10). The doses of SZSP-L, SZSP-M, and SZSP-H were 100, 200, and 400 mg/kg bw/d, respectively. Compared with the control group, ^#^*p* < 0.05 and ^##^*p* < 0.01. Compared with the model group, *p < 0.05 and **p < 0.01.

Histological analysis of the spleen is shown in Fig. 4a, b. H&E staining showed that the control group had a clear structure; lymphocytes, red pulp, and white pulp were regular and with clear edges. Compared with the control group, the white pulp of the CTX group was blurred and the area became smaller, while lymphocytes were sparse and their number decreased. Compared with the control group, the CTX group showed irregular arrangement of cells, unclear marginal boundary, obvious intercellular space dilatation, and an unclear red and white pulp structure. Compared with the CTX group, the state of spleen lobules was better, and the boundary between the cortex and medulla was clear in the SZSP-L (-M, -H), LH, PGI, and SPI groups. The number of lymphocytes increased, and the lymphocytes were round. These results showed that SZSP effectively prevented the CTX-related damage and performed its immunomodulatory functions by affecting the immune organs (Fig. 4a).

The immunohistochemical results of the spleen showed that the expression level of CD_4_^+^ in splenocytes of the model group was significantly lower than that of the control group. The expression level of CD_4_^+^ in splenocytes of each administration group significantly increased, while the expression level of CD_4_^+^ in the SZSP treatment group increased in a dose-dependent manner. The expression level of CD_8_^+^ in splenocytes of the mouse model group was significantly higher than that of the control group (*p* < 0.01). Compared with the control group, the expression level of CD_8_^+^ in spleen lymphocytes of the LH and SZSP groups decreased significantly after administration (Fig. 4b). These results further confirmed the protective effect of SZSP on the spleen.

### Effect of SZSP on cytokines of LPS-stimulated RAW264.7 cells

**Fig. 5:**
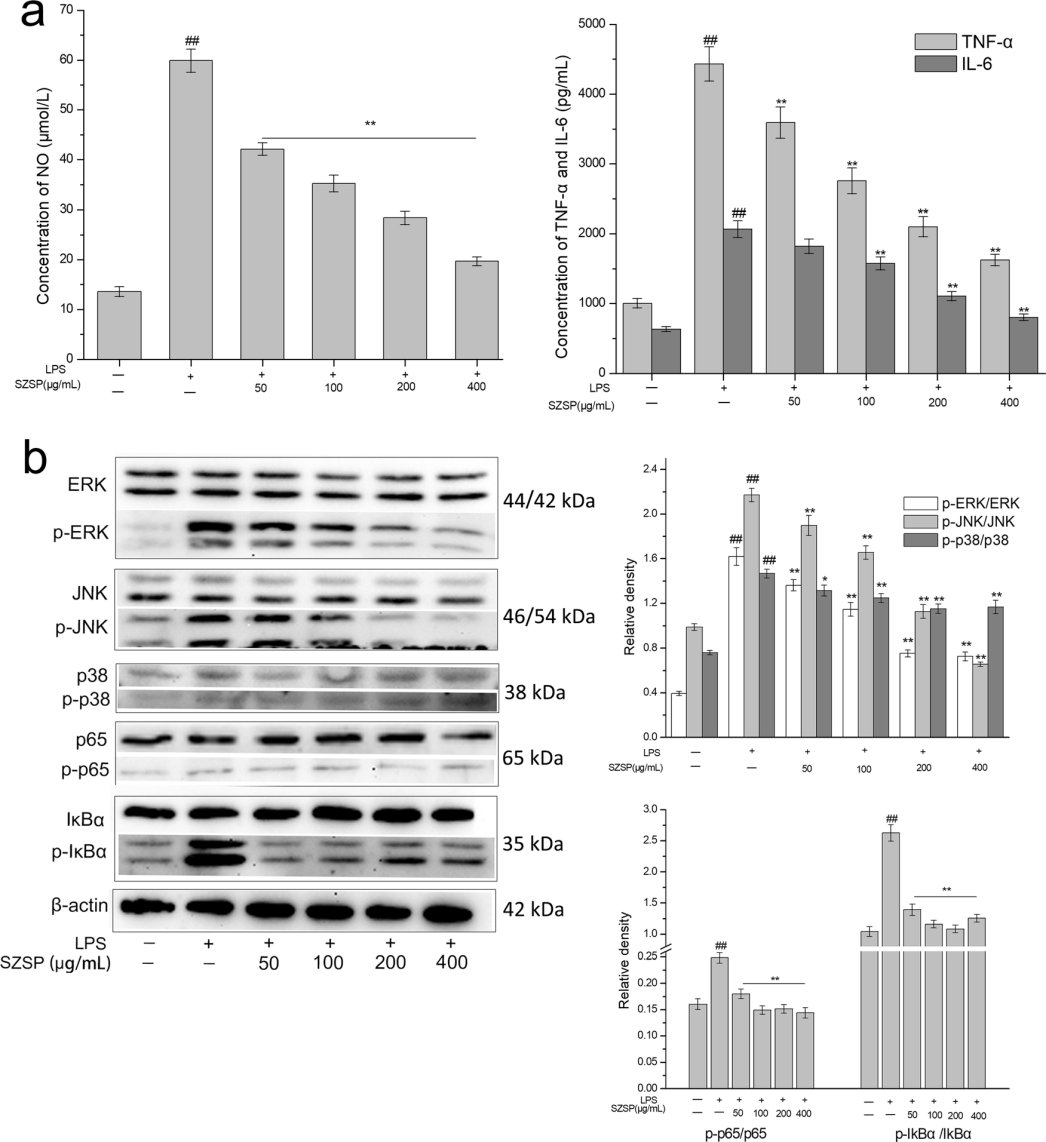
Effects of semen Ziziphi Spinosae protein (SZSP) on the immune regulation in LPS-stimulated RAW264.7 cells. **a** Effect of SZSP on NO, TNF-α, and IL-6 secretion in LPS-stimulated RAW264.7 cells. **b** Effect of SZSP on the expression levels of proteins related to the MAPKs and NF-κB pathways in LPS-stimulated RAW264.7 cells. The protein expression levels of ERK, p-ERK, JNK, p-JNK, p38, p-p38, p65, p-p65, IκBα, and p-IκBα in LPS-stimulated RAW264.7 cells were measured by western blot analysis. The results are shown as means ± SD (*n* = 3). The doses of SZSP were 50, 100, 200, and 400 μg/mL. Compared with the control group, ^#^*p* < 0.05 and ^##^*p* < 0.01. Compared with the LPS group, *p < 0.05 and **p < 0.01.

As shown in Fig. 5a, compared with the control group, the content of NO, IL-6, and TNF-α in RAW264.7 cells increased significantly under the stimulation with LPS (*p* < 0.01). With the intervention of SZSP, the cellular immunostimulatory response induced by LPS was inhibited, and the expression levels of NO, IL-6, and TNF-α decreased gradually in a dose-dependent manner.

### Effect of SZSP on the MAPKs and NF-κB pathways of LPS-stimulated RAW264.7 cells

MAPKs and NF-κB are the key pathways of immune function. They can stimulate the immune defense response in case of external stimuli, which includes extracellular signal-regulated kinase (ERK), c-Jun N-terminal kinase (JNK), p38, p65, and IκBα^31^. As shown in Fig. 5b, the expression levels of p-ERK, p-JNK, and p-p38 were significantly enhanced by LPS stimulation. SZSP treatment weakened the stimulation with LPS and reduced phosphorylation in RAW264.7 cells in a dose-dependent manner. According to the results of the SZSP-M group, the levels of p-ERK, p-JNK, and p-p38 decreased by 2.1-, 1.9-, and 1.3-fold, respectively. Similar results were found in the NF-κB pathway. LPS stimulation increased the expression of p-p65 and p-IκBα. With the intervention of SZSP, the phosphorylation level decreased, indicating that SZSP was able to inhibit LPS-stimulated immune stimulation. These results suggest that the immune enhancement of SZSP can be achieved by activating the MAPKs and NF-κB pathways.

### Purification and separation of SZSP

**Fig. 6:**
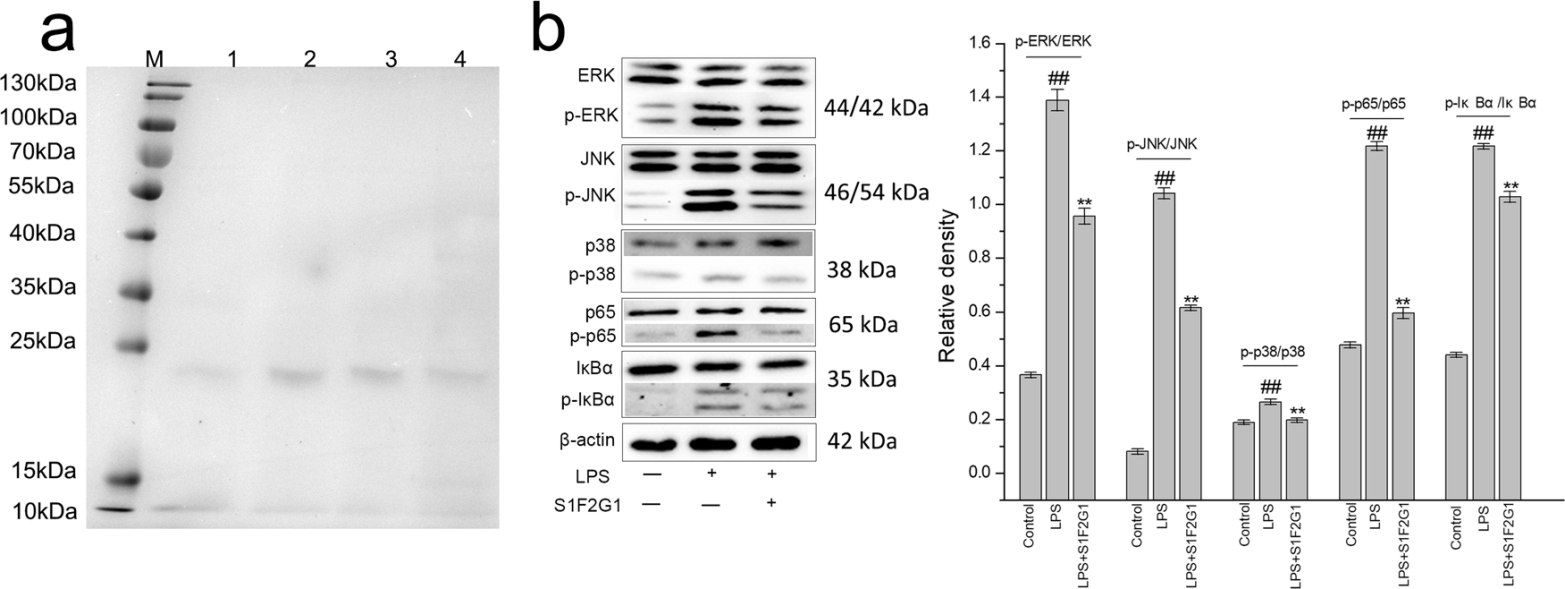
Effects of semen Ziziphi Spinosae protein (SZSP) separated components on the immune regulation in LPS-stimulated RAW264.7 cells. **a** SDS-PAGE of SZSP separated components (M: Marker, 1–4: S1F2G1 elution peak). **b** Effect of S1F2G1 on the expression levels of proteins related to the MAPKs and NF-κB pathways in LPS-stimulated RAW264.7 cells. The protein expression levels of ERK, p-ERK, JNK, p-JNK, p38, p-p38, p65, p-p65, IκBα, and p-IκBα in LPS-stimulated RAW264.7 cells were measured by western blot analysis. The results are shown as means ± SD (*n* = 3). The dose of SZSP was 200 μg/mL. Compared with the control group, ^#^*p* < 0.05 and ^##^*p* < 0.01. Compared with the LPS group, *p < 0.05 and **p < 0.01.

To further identify the components with significant immunomodulatory activity in SZSP, we conducted preliminary separation and purification experiments on SZSP on the basis of the above experimental studies. As shown in Supplementary Fig. 1, after Sephadex G-50 gel chromatography, there were four elution peaks, named S1–S4. They were collected and combined with each peak eluent and freeze-dried. The S1 protein component was adsorbed on the DEAE Sepharose FF chromatography column. Under the gradient elution of 0–1.0 mol/L NaCl buffer, there were five obvious absorption peaks, and each protein purification component was collected and named as component S1F1–S1F5. We integrated the solution collection, dialysis, and freeze-drying. After second Sephadex G-50 chromatography of S1F2 component, two elution peaks appeared, and they were named components S1F2G1 and S1F2G2. The eluents of each peak were collected and combined. After freeze-drying, the elution peaks were detected by SDS-PAGE (Fig. 6a). The results showed that the peaks of S1F2G1 and S1F2G2 were obvious, which were single bands and were the target products of SZSP separation and purification.

### MAPKs and NF-κB pathway detection of purified protein components

As shown in Fig. 6b, compared with the LPS-stimulated model group, the intervention with S1F2G1 component was able to significantly reduce the LPS-related stimulation of RAW264.7 cells, inhibit the phosphorylation of factors in the MAPKs and NF-κB signaling pathways, and enhance the cellular immune activity. Therefore, the purified S1F2G1 fraction had good immunoregulatory activity, which provided a preliminary indication for the further study of SZSP.

## Discussion

In recent years, with the rapid increase in the population with immune diseases, the problem of immune regulation has attracted the attention of researchers^32^. Immune regulation homeostasis is an unstable critical state that may change positively or negatively at any time. In this state, the body is particularly vulnerable to the invasion by viruses and bacteria, causing immunosuppressive and immune stimulation reaction, and even further producing related immune diseases^33^. The search for more effective immunomodulators has become the focus of many studies. With the deepening of research, protein components with immune-regulation properties obtained from natural plants have gradually attracted the interest of researchers. Plant protein resources are abundant, their production cost is low, and they have high nutritional value and fewer side effects^34,35^. Thus, they can meet both the functional characteristics of human daily consumption and industrial production. It is worth noting that with the deepening of research and the discovery of new plant protein sources, some plant proteins have also shown new biological activities. Some examples include the antioxidant ability of perilla seed protein, the antibacterial ability of Bauhinia seedling protein, and the antihypertensive activity of pumpkin seed protein^36–38^. Thus, plant proteins can be used as the main raw material source of functional food to improve immunity, and they have high scientific research value. Proteins from a variety of common foodborne plants also have excellent immunomodulatory effects. For example, whey protein can enhance ConA-induced splenocyte proliferation and immune factor secretion in mouse body fluid and serum^39^. Glycoprotein in rice can inhibit the release of NO, TNF-α, IL-6, and IL-1β in RAW264.7 cells induced by LPS^40^. Positively charged peptides in soy protein can increase the proliferation of mouse spleen lymphocytes and the phagocytic activity of peritoneal macrophages^41^. Thus, high-quality protein resources have good potential to enhance immune regulation of the body. The SZSP involved in this study was extracted from SZS, which is both a food and a drug. However, there was no relevant research report on the immunoregulatory activity of SZSP. Given that SZSP is a new source of plant protein with excellent processing properties in the food industry, it is necessary to comprehensively evaluate its immunomodulatory activity. Therefore, in this study, we comprehensively evaluated the immunomodulatory activity of SZSP through the experimental verification in vivo and in vitro. In addition, different extraction methods of SZSP were verified and analyzed. The pre-experimental analysis results showed that AA-SZSP obtained by AA had better extraction efficiency and immunomodulatory activity compared with AE and AS.

Immunity is an important physiological function of the body. The immune system of the human body is a complex and precise system and is composed of immune organs, immune cells, and immune active substances^42^. They coordinate with each other to play a role in biological immune regulation and protect the body from viruses, bacteria, and other foreign invasion^43^. The spleen and the thymus are the main immune organs of the human body. The immune organ index is usually used as the immune index of the body^44^. In immune research, the immunosuppression model induced by CTX has often been used as the basis for detecting immunosuppression. Therefore, we investigated the resistance of mice to immunosuppression after long-term oral administration of SZSP and further clarified the immunomodulatory advantages of SZSP by adding representative plant proteins (PGI and SPI) as positive controls. The results showed that SZSP was able to improve the morphology of immune organs, the phagocytic ability of peritoneal macrophages, NK-cell activity, and immune factor secretion in mice. The changes in the spleen- and thymus-related indexes reflect the overall immune situation. The proliferation of spleen cells, the ratio of CD_4_^+^/CD_8_^+^ T cells in the spleen and thymus, and immunoglobulins such as IgA, IgG, and IgM in the serum are the main components of the body’s immune system^45^. Peritoneal macrophages can phagocytize and digest viruses and bacteria and activate other immune cells and make them respond to pathogens and release a variety of immune factors, such as NO, IL-1β, IL-6, and TNF-α^46^. NK cells can secrete immune factors and are important immune cells in the body. They are not only involved in antitumor, antiviral, and immune regulation but also participate in hypersensitivity and autoimmune diseases in some cases^47^. In this study, after CTX induction, the spleen and the thymus in each of the administration group shrank and the organ index decreased. Oral administration of SZSP significantly improved CTX-induced immunosuppression of the thymus and spleen and increased the organ index. In addition, SZSP significantly increased the production of NO, IL-1β, and TNF-α in peritoneal macrophages, increased the phagocytic rate of peritoneal macrophages, enhanced the cytotoxicity of NK cells to YAC-1 cells, and improved splenic lymphocyte proliferation. SZSP changed the inhibition of the secretion of IFN-γ, IL-2, IgE, IgA, and IgM in mouse serum. We also found that plant proteins had poor regulatory ability on IL-2 and IgA, and only SZSP-H significantly affected their secretion status. Immunohistochemical results showed that after intraperitoneal injection of CTX, the secretion of CD_4_^+^ in the immune organs of the mice decreased, and the secretion of CD_8_^+^ increased. Generally speaking, CD_4_^+^ lymphocytes decreased, which is the main development trend of malignant tumors, immunodeficiency diseases, and AIDS. The increase in CD_8_^+^ lymphocytes is the main reference index for the detection of autoimmune diseases. The CD_4_^+^/CD_8_^+^ ratio reflects the strength of the immune regulation ability. When the ratio decreases gradually, it indicates that the immune ability of the body is weaker. Therefore, in this study, SZSP was able to regulate the secretion of CD_4_^+^ and CD_8_^+^ in the immune organs and restore CD_4_^+^/CD_8_^+^ to the normal proportion range, indicating that SZSP has good immune regulation ability in vivo. These results suggest that SZSP can effectively enhance innate and adaptive immunity in CTX-induced immunosuppressed mice.

Macrophages play a unique role in the immune system and can secrete many regulatory factors, such as NO, TNF-α, and IL-6, to regulate the immune balance of the body. In in-vitro studies, RAW264.7 macrophages are often stimulated with LPSs to achieve the purpose of immune stimulation^48^. LPS is the endotoxin component of Gram-negative bacteria. It has a significant effect on TLR4 in Toll-like receptor and activates NF-κB, MAPKs, and other signaling pathways, resulting in the excessive production of immune mediators and cytokines, and then activates the body’s immune response^49^. Thus, LPS-activated RAW264.7 macrophages are widely used as an immunostimulatory cell model. In view of the immunomodulatory effect of SZSP on CTX-induced immunosuppressive mice, its specific mechanism has not been explored. Therefore, we examined the effect of SZSP on the MAPKs and NF-κB signaling pathways in the LPS-stimulated RAW264.7 cell model so as to explore its potential immunomodulatory mechanism. In this work, based on the LPS-stimulated RAW264.7 model, the negative immunoregulatory effect of SZSP was evaluated by the levels of NO, TNF-α, and IL-6 production, and the mechanism of major factors in the MAPKs and NF-κB signaling pathways was detected by western blot analysis. Consequently, as expected, we showed that SZSP significantly reduced the levels of p-ERK, p-JNK, and p-IκBα and activated the MAPKs and NF-κB signaling pathways, thereby improving immunosuppression and activating host defense. To further confirm the components with immunoregulatory activity in SZSP, SZSP was purified by gel chromatography, and the S1F2G1 component with a single protein electrophoresis band was extracted and separated. Western blot analysis showed that S1F2G1 had good immunoregulatory activity, meaning that S1F2G1 may be an important active component of SZSP. These results provide a basic reference for further component analysis and pharmacological activity study of SZSP and suggest new research ideas and directions.

In summary, this study reports and proves the immunomodulatory activity of SZSP, providing insights into the development of SZSP products in enhancing immunomodulatory ability and health improvement. SZSP exhibited immunostimulatory effects in CTX-induced immunosuppressed mice by enhancing the immune organ development, the phagocytosis of peritoneal macrophages and improving the expression of T lymphocyte subsets simultaneously, ultimately alleviating the body state of immunosuppressed mice. In addition, SZSP can also affect the phosphorylation expression of p-ERK, p-JNK, and p-IκBα in the NF-κB and MAPKs signaling pathways, and preliminarily elucidate the key mechanism of immunomodulatory effect. The purified S1F2G1 component also exhibited significant immunomodulatory activity, further proving the development potential of SZSP in regulating immunosuppression. Therefore, in the field of functional foods, SZSP may be a promising candidate for regulating immune responses.

## Methods

### Materials and reagents

SZSP was extracted in line with our previous research methods (protein content: 83.45 ± 0.85%)^20^, and the raw materials were from the Affiliated Hospital of Changchun University of Chinese Medicine. CTX (S30563) and levamisole hydrochloride (LH, S30366) were purchased from Yuanye Bio-Technology Co. (Shanghai, China). *Panax ginseng* C. A. Meyer protein (PGI) was extracted in line with the published report, and the raw material was from the Affiliated Hospital of Changchun University of Traditional Chinese Medicine. Soy protein isolate (SPI, S9510) was obtained from Solarbio Science & Technology Co., Ltd. (Beijing, China). RAW264.7 (a murine macrophage/monocyte-like cell line, C7505) cells and lymphoblast YAC-1 (CBP60886) cells were obtained from the cell bank of the Chinese Academy of Science (Shanghai, China). Nitric oxide (NO, S0023) assay kit was obtained from Beyotime Biotech. (Jiangsu, China). Lipopolysaccharide (LPS, L2880) was obtained from Sigma (St. Louis, MO, USA). ELISA kits of IL-1β (SEA563Mu), IL-6 (SEA079Mu), TNF-α (SEA133Mu), IL-4 (SEA077Mu), and IFN-γ (SEA049Mu) were purchased from Cloud-Clone Corp. (Wuhan, China). Antibodies against phospho-p65 (ab32536), IκBα (ab32518), phospho-p38 (ab4822), phospho-JNK (ab124956), phospho-ERK (ab201015), conjugated anti-mouse CD_4_ (ab183685), APC-conjugated anti-mouse CD_8_ (ab217344) antibodies, and horseradish peroxidase (HRP)–conjugated goat anti-rabbit immunoglobulin (Ig) G were purchased from Abcam plc. (Cambridge, UK). ECL chemiluminescence kit (P0018S) was purchased from Beyotime Biotech. Inc. (Beijing, China).

### Comparison of immunomodulatory activities of different protein extraction methods

Different common methods for edible plant protein extraction were used to extract the protein from SZS. The following three protein extraction methods were comprehensively evaluated using the LPS-stimulated RAW264.7 cell model experiment and protein extraction rate. The specific methods were as follows:

*Ammonium sulfate precipitation method (AS-SZSP)*: Defatted SZS flour was mixed with 0.01 M phosphate buffer (PB, pH 7.0) at a ratio of 1:10 (w/v, g/mL). The mixtures were stored at 4 °C and stirred for 12 h. The supernatant was collected by centrifugation at 5000 rpm for 15 min at 4 °C. A final concentration of 80% saturated ammonium sulfate was added to the supernatant. After centrifugation (4 °C, 5000 rpm, 15 min), the precipitated protein was dissolved in distilled water and dialyzed for 48 h at 4 °C. Then, it was stored at −20 °C after freeze-drying^50^.

*Alkali extraction and acid dissolution method (AA-SZSP)*: Defatted SZS flour was dispersed in pure water (1:20, w/v), and the pH of the suspension was adjusted to 9.5 with 1 M NaOH. The suspension was continuously stirred at 50 °C for 45 min and then centrifuged at 3500 rpm for 15 min to collect the supernatant. Then, we added 1 M HCl, adjusted the supernatant to the isoelectric point of 4.2, and then incubated it at 4 °C for 2 h. We centrifuged it at 3500 rpm for 10 min to collect the precipitate. The precipitated protein was resuspended in distilled water, and 1 M NaOH was added to adjust the pH to 7.0. The suspension was freeze-dried and stored at 4 °C^51^.

*Aqueous extraction method (AE-SZSP)*: Defatted SZS flour was diluted in water, fully mixed and stirred at room temperature for 30 min, and pH was adjusted, followed by stirring for 10 min. The combination was centrifuged at 5000 rpm for 10 min, and the pH of the obtained supernatant was adjusted to 4.3 using 1 M HCl. Again, after centrifugation at 5000 rpm for 10 min, the precipitates were rinsed with deionized water. The protein isolate was stored at −20 °C after lyophilization^52^.

### Animals and experimental design

Six-week-old male ICR mice (18–22 g) were obtained from Liaoning Changsheng Biotechnology Co., Ltd. (Shenyang, China) and housed under standard laboratory conditions, i.e., light/dark light cycle: 12 h/12 h, temperature: 22–25 °C, and relative humidity: 55% ± 10%. All mice were handled in strict adherence with the Rules for Medical Laboratory Animals of the Ministry of Health, China. The Animal Care & Welfare Committee of Changchun University of Chinese Medicine approved all of the procedures, and the approval number was 20200328. After a 7-day acclimatization period, the mice were randomly divided into eight groups (*n* = 15) as follows: control group (untreated, normal saline orally); model group (CTX group, cyclophosphamide); three SZSP groups, namely, SZSP-L (low-dose group, 100 mg/kg bw/d), SZSP-M (medium-dose group, 200 mg/kg bw/d), and SZSP-H (high-dose group, 400 mg/kg bw/d); LH group (positive control, levamisole hydrochloride, 10 mg/kg bw/d); and two protein positive control groups, namely, PGI and SPI (200 mg/kg bw/d)^10,53^.

The mice were orally administered with LH, SZSP, SPI, and PGI for 18 consecutive days. On the 15th day, the mice in all of the groups except the control group were intraperitoneally injected with 80 mg/kg CTX for 3 days. The body weights of the mice were monitored daily. Calculation of the organ index was based on the changes in body weight as follows: index (mg/g) = (weight of thymus or spleen)/body weight.

### Levels of serum cytokines by ELISA

Serum was obtained from eyeball blood collected by centrifugation at 3000 rpm for 15 min. Levels of IL-4, IFN-γ, IgA, IgM, and IgG were detected by ELISA kits.

### Isolation of peritoneal macrophages and splenic lymphocytes

Splenic lymphocytes and peritoneal macrophages were prepared using Tang method with minor modifications^54^. Specifically, 5 mL phosphate-buffered saline (PBS, ice-cold for 4 °C) was injected into the peritoneal cavity of the mice and finger-massaged for 5 min, and macrophages were aseptically collected. Splenic lymphocytes were obtained by taking out the excised spleen and grinding it gently in the filter screen to obtain single-cell suspension. We added red cell lysis buffer, mixed well for 5 min, and collected splenocytes by centrifugation.

### Levels of cytokines secreted by peritoneal macrophages

The peritoneal macrophages were seeded at a density of 1 × 10^6^ cells/well in 96-well plates, and the supernatant was collected after 48 h. Levels of TNF-α, IL-2, and IL-6 in the supernatants were determined using ELISA kits, and NO concentration was measured by nitric oxide assay kit, following the manufacturer’s instructions. Apoptosis of the primary peritoneal macrophages was detected by flow cytometry.

### Phagocytosis of peritoneal macrophages

Phagocytosis of macrophages was measured by the neutrophil proliferation and cytotoxicity test kit. Specifically, peritoneal macrophages were seeded at a density of 1 × 10^6^ cells/well in the 96-well plate and cultured for 24 h. Neutral red detection solution (20 μL/well) was added and incubated for 60 min. The cells were washed twice with PBS cleaning solution; neutral red cracking solution was added and maintained at room temperature for 10 min; and the absorbance value was detected at 540 nm.

### Proliferation and NK cell cytotoxicity of splenic lymphocytes

The proliferation of splenic lymphocytes was detected by CCK-8 assays after induction by Con A (5 µg/mL) and LPS (10 µg/mL). Briefly, the isolated splenic lymphocytes were diluted to the concentration of 3 × 10^5^ cells/mL using RPMI-1640 medium containing fixed concentrations of Con A and LPS. Then, 100 µL cell suspension was seeded into each well of a 96-well plate, and was detected with a CCK-8 kit after 24 h of culture.

NK-cell activity was analyzed by co-culture of splenic lymphocytes (effector cells) with YAC-1 cells (target cells) (Monmai et al., 2019). After co-incubation of splenocytes (2 × 10^5^ cells/100 μL/well) and YAC-1 cells (1 × 10^4^ cells/100 μL/well) in a 96-well plate for 24 h, the cells were subjected to the CCK-8 assay. RPMI-1640 medium was used as control.

### Levels of serum hemolysin

The serum hemolysin test was performed as described by Bin Hafeez et al., with some modifications^55^. On the 15th day, all of the mice were intraperitoneally injected with 200 μL of sheep red blood cell suspension (SRBC) (10^9^ cells/mL). Twenty-four hours after the last administration, the blood of the mice in each group was collected. Mouse serum was extracted by centrifugation at 2000 rpm for 15 min, and it was diluted with normal saline (ratio 1:200). Then, we added 500 μL of 10% SRBC and 500 μL of 10% fresh guinea pig serum to the dilution. After incubation at 37 °C for 1 h, the absorbance was measured at 540 nm.

### Histopathological analysis

After the weight measurement of the mice, the spleen and the thymus were aseptically separated and fixed in 10% formalin solution for 24 h. Then, after programmed dehydration, paraffin-embedded sections were prepared and 4-μm-thick sections were cut. The tissue sections were stained with hematoxylin and eosin (H&E) and observed under an optical Nikon microscope (Tokyo, Japan).

### Immunohistochemistry

After dewaxing and rehydration, the tissue sections were boiled in 10 mM sodium citrate buffer to recover the antigen. Endogenous peroxidase was inactivated with 3% H_2_O_2_ for 30 min, and nonspecific immunoglobulin binding was blocked with normal goat serum. After blocking, the sections were incubated with CD_4_^+^ (1:100) and CD_8_^+^ (1:100) antibodies at 4 °C. After washing with PBS, the sections were incubated with secondary anti-goat antibody at room temperature for 1 h. The sections were then stained using an avidin/biotinylase complex (ABC) kit. Then, the sections were observed under an optical Nikon microscope (Tokyo, Japan).

### Cell culture and cytokines of RAW264.7 cells

RAW264.7 cells were cultured in Dulbecco’s modified Eagle medium (DMEM) supplemented with 10% (v/v) fetal bovine serum and 1% penicillin/streptomycin. All cells were maintained in a humidified incubator with 5% CO_2_ at 37 °C. All experimental studies were performed when the cells were in the logarithmic growth phase. The levels of NO, TNF-α, and IL-6 in the supernatant were measured using Griess and ELISA method. Briefly, RAW264.7 cells in the logarithmic growth phase were taken, and the dilution concentration was 1 × 10^6^ cells/mL. The cells were incubated in 96-well plates for 24 h. Then, SZSP at different concentrations (50, 100, 200, 400 μg/mL) and LPS (1 μg/mL) were added to the cultured cells in each well and incubated for 24 h. The detection was carried out in line with the standard operating instructions of Griess and ELISA kit.

### Western blot analysis

The protein expression of SZSP activation pathway in RAW264.7 cells was evaluated by western blot analysis. Proteins were prepared from RAW264.7 cells using a total protein extraction kit in line with the manufacturer’s instructions. The protein concentration was determined by using a BCA protein detection kit. The denatured protein was isolated by 10% SDS-PAGE and then transferred to polyvinylidene fluoride (PVDF) membranes. After blocking with 5% skimmed milk at room temperature for 60 min, the membranes were mixed with primary antibody (p-NF-κB p65, p-IκBα, P-p38, p-ERK, p-JNK, diluted 1:1000) and incubated overnight at 4 °C. The membranes were incubated with secondary antibody at room temperature for 60 min (1:1000 dilution). Protein bands were detected using ECL kit, and signals were visually analyzed using Bio-Rad image analysis system (Bio-Rad, CA, USA).

### Purification of SZSP

SZSP was isolated and purified using an Akta avant (GE Healthcare, Marlborough, USA), and the gel column was packed with Sephadex G50 (G50) and DEAE-Sepharose Fast Flow (FF). The SZSP detection solution (20 mg/mL) was prepared with 20 mM Tris-HCl buffer (pH 7.4). The FF gel column program was set at 20 mmol/L Tris-HCl (pH 7.4) and 1 mol/L NaCl (pH 7.4). The concentration curve was (0–100%, 0–120 min), and elution velocity was 5 mL/min. The separated protein fractions were filtered to remove salts using a G50 gel column (Marlborough, USA) and ultrapure water, and the eluted proteins were collected and freeze-dried.

### Statistical analysis

In this study, the statistical analyses were performed using SPSS software (version 21.0, SPSS Inc, Chicago, IL, USA) and Origin software (version 8.0, Origin Lab, CA, USA). The data were obtained by conducting at least three independent experiments and were expressed as the mean ± SD. The one-way ANOVA and *t* tests were used for the statistical significance analysis. *P* values lower than 0.05 were considered statistically significant.

### Reporting summary

Further information on research design is available in the Nature Research Reporting Summary linked to this article.

## Supplementary information

Supplemental Material

Reporting Summary

## Data Availability

The data supporting the findings reported herein are available on reasonable request from the corresponding author.
