# Supplementary material for: Immunomodulatory activity of semen Ziziphi Spinosae protein: a potential plant protein functional food raw material
Source: NPJ Sci Food. 2023 Jun 19;7:32. doi: 10.1038/s41538-023-00204-3 (PMC10279729; doi:10.1038/s41538-023-00204-3)

Supplementary figure 1 Isolation and purification of SZSP.

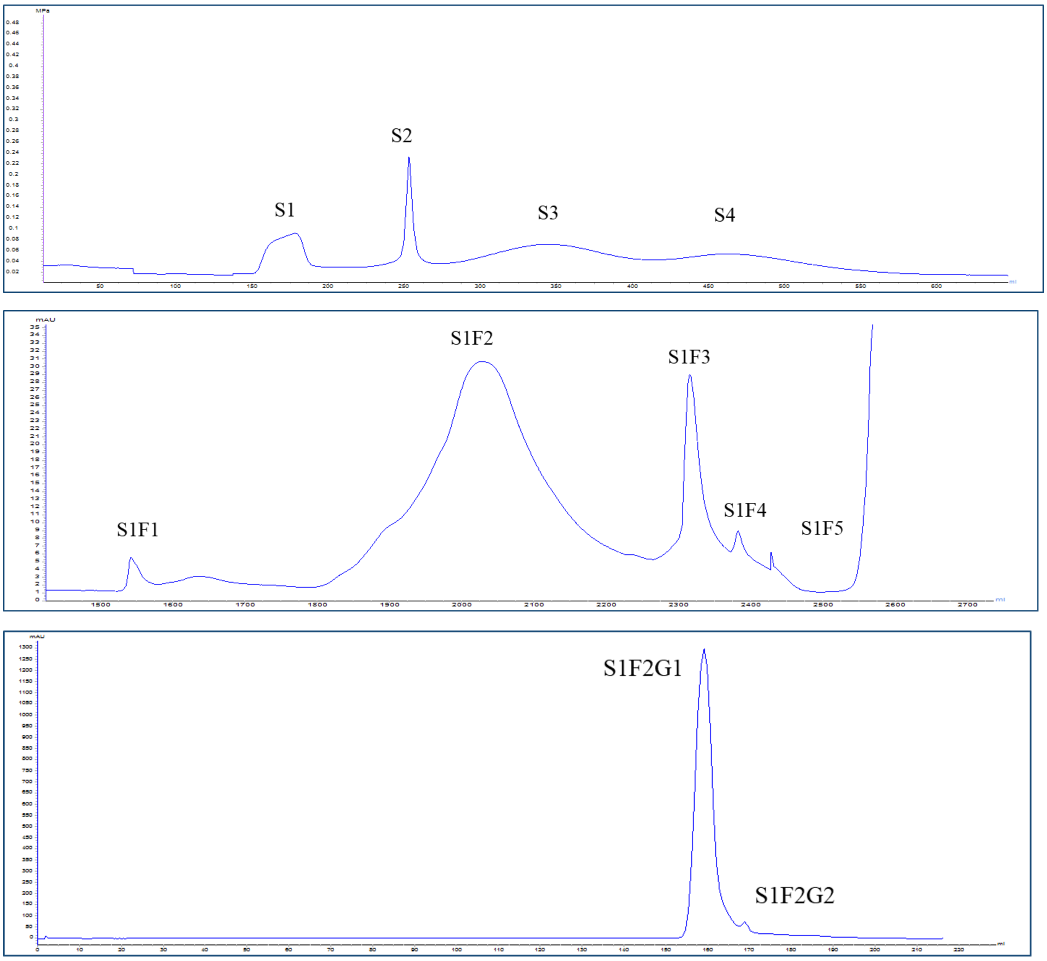

SDS-PAGE detection of the total protein extraction from RAW264.7 cells

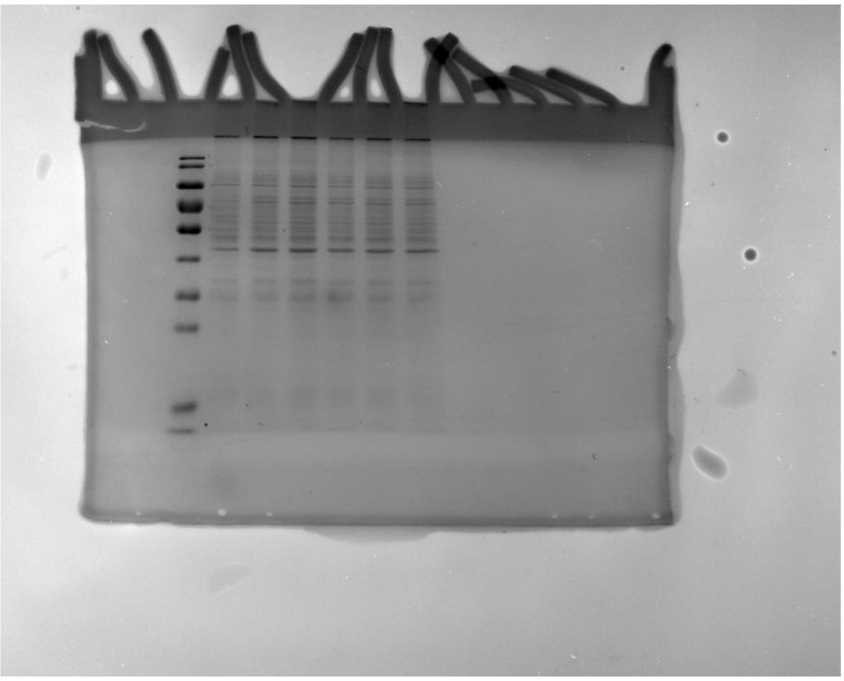

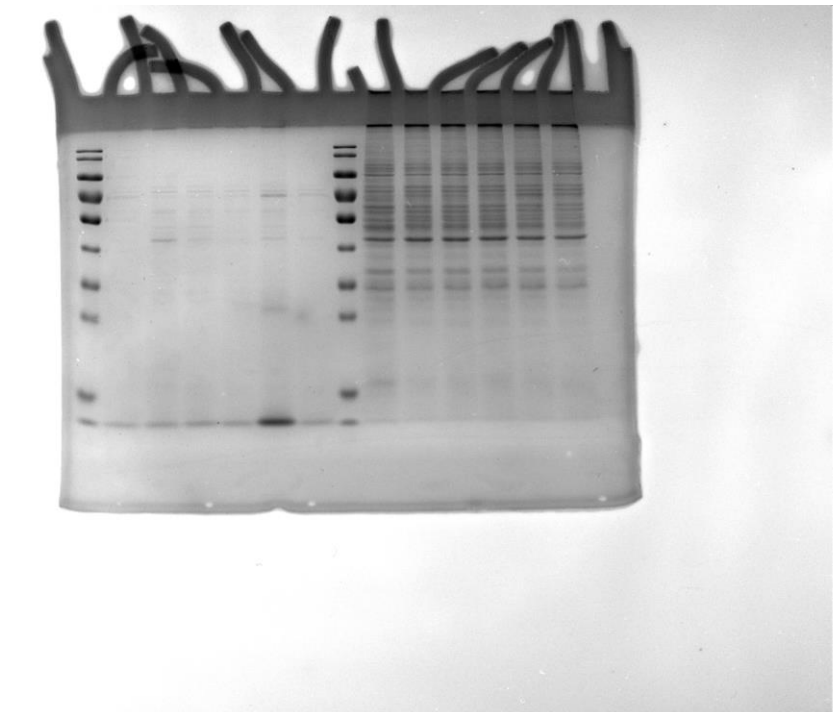

Supplement: Supplementary file 1 — Supplemental Material [file 41538_2023_204_MOESM1_ESM.pdf]
