# Supplementary material for: Immunomodulatory activity of semen Ziziphi Spinosae protein: a potential plant protein functional food raw material
Source: NPJ Sci Food. 2023 Jun 19;7:32. doi: 10.1038/s41538-023-00204-3 (PMC10279729; doi:10.1038/s41538-023-00204-3)
Supplement: Supplementary file 2 — Reporting Summary [file 41538_2023_204_MOESM2_ESM.pdf]

nature portfolio

Corresponding author(s): Mingming Yan, NPJSCIFOOD-00647R

Last updated by author(s): May 6, 2023

Reporting Summary

Nature Portfolio wishes to improve the reproducibility of the work that we publish. This form provides structure for consistency and transparency in reporting. For further information on Nature Portfolio policies, see our Editorial Policies and the Editorial Policy Checklist.

Statistics

For all statistical analyses, confirm that the following items are present in the figure legend, table legend, main text, or Methods section.

n/a	Confirmed	
☐	☒	The exact sample size (n) for each experimental group/condition, given as a discrete number and unit of measurement
☐	☒	A statement on whether measurements were taken from distinct samples or whether the same sample was measured repeatedly
☐	☒	The statistical test(s) used AND whether they are one- or two-sided<br>*Only common tests should be described solely by name; describe more complex techniques in the Methods section.*
☐	☒	A description of all covariates tested
☐	☒	A description of any assumptions or corrections, such as tests of normality and adjustment for multiple comparisons
☐	☒	A full description of the statistical parameters including central tendency (e.g. means) or other basic estimates (e.g. regression coefficient) AND variation (e.g. standard deviation) or associated estimates of uncertainty (e.g. confidence intervals)
☐	☒	For null hypothesis testing, the test statistic (e.g. F, t, r) with confidence intervals, effect sizes, degrees of freedom and P value noted<br>*Give P values as exact values whenever suitable.*
☐	☒	For Bayesian analysis, information on the choice of priors and Markov chain Monte Carlo settings
☐	☒	For hierarchical and complex designs, identification of the appropriate level for tests and full reporting of outcomes
☐	☒	Estimates of effect sizes (e.g. Cohen's d, Pearson's r), indicating how they were calculated

Our web collection on statistics for biologists contains articles on many of the points above.

Software and code

Policy information about availability of computer code

Data collection	Researchers recorded the weight of mice and other experimental data using Microsoft Office Software (Excel) 2020, which was activated by the original version.
Data analysis	Researchers used SPSS software (version 21.0, SPSS Inc, Chicago, IL, USA) and Origin software (version 8.0, Origin Lab, CA, USA) to analyze and calculate the experimental data. The above software is free and open source software.

For manuscripts utilizing custom algorithms or software that are central to the research but not yet described in published literature, software must be made available to editors and reviewers. We strongly encourage code deposition in a community repository (e.g. GitHub). See the Nature Portfolio guidelines for submitting code & software for further information.

Data

Policy information about availability of data

All manuscripts must include a data availability statement. This statement should provide the following information, where applicable:

- Accession codes, unique identifiers, or web links for publicly available datasets
- A description of any restrictions on data availability
- For clinical datasets or third party data, please ensure that the statement adheres to our policy

The data supporting the findings reported here are available on reasonable request from the corresponding author.

Research involving human participants, their data, or biological material

Policy information about studies with human participants or human data. See also policy information about sex, gender (identity/presentation), and sexual orientation and race, ethnicity and racism.

Reporting on sex and gender	This study does not involve this item.
Reporting on race, ethnicity, or other socially relevant groupings	This study does not involve this item.
Population characteristics	This study does not involve this item.
Recruitment	This study does not involve this item.
Ethics oversight	This study does not involve this item.

Note that full information on the approval of the study protocol must also be provided in the manuscript.

Field-specific reporting

Please select the one below that is the best fit for your research. If you are not sure, read the appropriate sections before making your selection.

☒ Life sciences ☐ Behavioural & social sciences ☐ Ecological, evolutionary & environmental sciences

For a reference copy of the document with all sections, see nature.com/documents/nr-reporting-summary-flat.pdf

Life sciences study design

All studies must disclose on these points even when the disclosure is negative.

Sample size	In this study, the sample size is determined according to the specific experimental data requirements and relevant literature reports.
Data exclusions	There is no data exclusion in this study
Replication	In this study, after long-term feeding and modeling of mice, no data difference was found to be too large and unreasonable. All results were within the reasonable range. The author has marked the error bar in the manuscript.
Randomization	In this study, mice were randomly assigned to each experimental group after 7 days of adaptive feeding. In addition, the distribution of test kits and cell experiments are randomly generated.
Blinding	In this study, all experimental data were obtained by real detection, and data analysis was carried out according to the original experimental design. The data detection of all groups were randomly selected, and complied with the basic rules of scientific research.

Reporting for specific materials, systems and methods

We require information from authors about some types of materials, experimental systems and methods used in many studies. Here, indicate whether each material, system or method listed is relevant to your study. If you are not sure if a list item applies to your research, read the appropriate section before selecting a response.

Materials & experimental systems

n/a	Involved in the study	
☐	☒	Antibodies
☐	☒	Eukaryotic cell lines
☒	☐	Palaeontology and archaeology
☐	☒	Animals and other organisms
☒	☐	Clinical data
☒	☐	Dual use research of concern
☐	☒	Plants

Methods

n/a	Involved in the study	
☒	☐	ChIP-seq
☒	☐	Flow cytometry
☒	☐	MRI-based neuroimaging

Antibodies

Antibodies used	Antibodies against phospho-p65 (ab32536), IκBα (ab32518), phospho-p38 (ab4822), phospho-JNK (ab124956), phospho-ERK

Antibodies used	(ab201015), conjugated anti-mouse CD4 (ab183685), APC-conjugated anti-mouse CD8 (ab217344) antibodies, and horseradish peroxidase (HRP)–conjugated goat anti-rabbit immunoglobulin (Ig) G were purchased from Abcam plc. (Cambridge, UK).
Validation	In this study, all antibodies are rabbit monoclonal antibodies with significant species reactivity with mice and RAW264.7 cells, and are applicable to WB, IHC-P, ICC/IF, IP and other analytical studies.

Eukaryotic cell lines

Policy information about cell lines and Sex and Gender in Research

Cell line source(s)	In this study, RAW264.7 cells (mouse mononuclear macrophage leukemia cells) were established by W. C. Raschke of the Salk Institute of California in the ascites of male BAB/14 mice induced by intraperitoneal injection of A-MuLV (Abelson murine leukemia virus), which is one of the most common cell models for immune research.
Authentication	In this study, RAW264.7 cells without any induction were used, which were round in shape and belonged to adherent cells. The specific identification of cells is performed by the cell company.
Mycoplasma contamination	The cells in this study were not contaminated by mycoplasma.
Commonly misidentified lines (See ICLAC register)	This study does not involve this item.

Animals and other research organisms

Policy information about studies involving animals; ARRIVE guidelines recommended for reporting animal research, and Sex and Gender in Research

Laboratory animals	In this study, The ICR mice (male, Six-week-old, 18–22 g) were obtained from Liaoning Changsheng Biotechnology Co., Ltd. (Shenyang, China).
Wild animals	In this study, ICR mice (male, Six-week-old, 18–22 g) were purchased by mice breeding companies with professional qualifications and national records. The mice are transported through aseptic treatment. Before entering the professional breeding room, the mice need to undergo further professional disinfection and sterilization to ensure that the mice are not contaminated by bacteria. At the end of the experiment, the mice were killed by decapitation and then treated by the university management department according to the unified requirements.
Reporting on sex	In this study, male ICR mice were selected for the main reason that they were consistent with the source of cell experiment. In addition, some studies have shown that male mice can achieve better analysis results for immunological studies.
Field-collected samples	The mice were housed under standard laboratory conditions, i.e., light/dark light cycle: 12 h/12 h, temperature: 22–25°C, and relative humidity: 55%±10%. All mice were handled in strict adherence with the Rules for Medical Laboratory Animals of the Ministry of Health, China.
Ethics oversight	The animal experiment scheme design of this study was approved by the Experimental Animal Ethics Committee of Changchun University of Traditional Chinese Medicine with the approval number of 20200328.

Note that full information on the approval of the study protocol must also be provided in the manuscript.

Dual use research of concern

Policy information about dual use research of concern

Hazards

Could the accidental, deliberate or reckless misuse of agents or technologies generated in the work, or the application of information presented in the manuscript, pose a threat to:

No	Yes	
☒	☐	Public health
☒	☐	National security
☒	☐	Crops and/or livestock
☒	☐	Ecosystems
☒	☐	Any other significant area

Experiments of concern

Does the work involve any of these experiments of concern:

No	Yes	
☒	☐	Demonstrate how to render a vaccine ineffective
☒	☐	Confer resistance to therapeutically useful antibiotics or antiviral agents
☒	☐	Enhance the virulence of a pathogen or render a nonpathogen virulent
☒	☐	Increase transmissibility of a pathogen
☒	☐	Alter the host range of a pathogen
☒	☐	Enable evasion of diagnostic/detection modalities
☒	☐	Enable the weaponization of a biological agent or toxin
☒	☐	Any other potentially harmful combination of experiments and agents
